# ^1^H Nuclear magnetic resonance based targeted profiling of metabolic responses induced by DNA damaging agents and PARP inhibition in MCF-7 cells

**DOI:** 10.1186/2049-3002-2-S1-P8

**Published:** 2014-05-28

**Authors:** Vijesh Bhute, Daniel Beard, Stephen Kron, Sean Palecek

**Affiliations:** 1 Chemical and Biological Engineering, University of Wisconsin-Madison, Madison, WI, USA; 2 Molecular and Integrative Physiology, University of Michigan, Ann Arbor, MI, USA; 3 Department of Molecular Genetics and Cell Biology, The University of Chicago, Chicago, IL, USA

## Background

Maintaining DNA integrity is essential for cell survival and proper functioning. Therefore, many cancer therapies, including radiotherapy and several chemotherapeutic drugs, induce damage to the DNA leading to genomic instability [1]. There are several DNA damage responses (DDRs) which can be activated depending on the type and the extent of DNA damage [1]. These responses involve detection of the damage by several proteins including, poly (ADP-ribose) polymerase (PARP), which can directly or indirectly affect metabolism [1].

## Materials and methods

We have used NMR based targeted profiling [2] to compare different DNA damage responses and study the effect of PARP inhibition on metabolism in the MCF-7 cell line. Methyl methanesulphonate (MMS), cisplatin, γ-radiation, and zeocin; were used to trigger different DDRs. ABT-888 (Veliparib) was used to study the effect of PARP inhibition on metabolism, both as a single agent and in combination with DNA damaging agents.

## Results

We observed several similarities in the metabolic responses caused by different DDRs and also identified specific metabolite markers on treatment with these DNA damaging agents. PARP inhibition restored most of the metabolic changes observed in MMS-treated cells suggesting that the metabolic response observed after MMS treatment may be predominantly due to PARP activation.

## Conclusions

In this study, we identified several similarities and differences in metabolic responses induced by different types of DDRs, which suggests the redirection of metabolic fluxes in a DNA damage-dependent manner. We also observed that recruitment of PARP during the DNA repair process can lead to widespread metabolic changes by observing the effect of PARP inhibition in MMS-treated cells. These results have identified novel metabolic responses in cancer cells following DNA damage and on inhibiting activity of proteins involved in DNA repair processes.
